# The transition from animal to human culture—simulating the social protocell hypothesis

**DOI:** 10.1098/rstb.2021.0416

**Published:** 2023-03-13

**Authors:** Claes Andersson, Tamás Czárán

**Affiliations:** ^1^ Department of Space, Earth and Environment, Division for Physical Resource Theory, Complex System Group, Chalmers University of Technology, 412 96 Gothenburg, Sweden; ^2^ European Centre for Living Technology, University of Venice Ca’ Foscari, Ca' Bottacin, Dorsoduro 3911, Calle Crosera, 30123 Venice, Italy; ^3^ Evolutionary Systems Research Group, ELKH Centre for Ecological Research, Karolina Road 29, H-1113 Budapest, Hungary; ^4^ Institute of Evolution, ELKH Centre for Ecological Research, Karolina Road 29, H-1113 Budapest, Hungary; ^5^ ELKH-ELTE Theoretical Biology and Evolutionary Research Group, Eötvös Loránd University, Egyetem tér 1–3, H-1053 Budapest, Hungary

**Keywords:** cultural evolution, origin of human culture, evolutionary transitions in individuality, simulation, social protocell, sociont

## Abstract

The origin of human cumulative culture is commonly envisioned as the appearance (some 2.0–2.5 million years ago) of a capacity to faithfully copy the know-how that underpins socially learned traditions. While certainly plausible, this story faces a steep ‘startup problem’. For example, it presumes that ape-like early *Homo* possessed specialized cognitive capabilities for faithful know-how copying and that early toolmaking actually required such a capacity. The social protocell hypothesis provides a leaner story, where cumulative culture may have originated even earlier—as cumulative *systems* of non-cumulative traditions ('institutions' and ‘cultural lifestyles'), via an emergent group-level channel of cultural inheritance. This channel emerges as a side-effect of a specific but in itself unremarkable suite of social group behaviours. It is independent of faithful know-how copying, and an ancestral version is argued to persist in *Pan* today. Hominin cultural lifestyles would thereby have gained in complexity and sophistication, eventually becoming independent units of selection (socionts) via a cultural evolutionary transition in individuality, abstractly similar to the origin of early cells. We here explore this hypothesis by simulating its basic premises. The model produces the expected behaviour and reveals several additional and non-trivial phenomena as fodder for future work.

This article is part of the theme issue ‘Human socio-cultural evolution in light of evolutionary transitions’.

## 1. Introduction

It has long been observed that social learning brings a potential for natural selection to act directly on cognitive and behavioural patterns rather than via the genetic basis of their neural substrate [1–4]. This Darwinian potential of culture points to a possible ‘dual-inheritance’ avenue for a transition from animal behaviour to human culture, via coevolution between biological and cultural units of selection. ‘Animal cultures’ play a central role in this story as an ancestral type of state where social learning gives rise to lineages of behaviour (traditions) that spread and persist in social networks across several generations.

However, while clearly ancestral, animal culture as such does not lead to something like human culture. To the contrary, animal culture has turned out to be a widespread and presumably ancient phenomenon (e.g. [5–7]). It was only in *Homo* that animal culture went on to transcend what otherwise amounts to a modest and dead-end extension of social learning with, at most, marginal Darwinian qualities [8].

Animal traditions may spread and persist, but in general they will not improve, and they will not become more complex over time [9–11]. Human culture, by contrast, undergoes open-ended cumulative evolution [12–15] whereby cultural know-how^1^ may be refined, inter-linked and expanded, seemingly indefinitely. The explanatory burden thereby moves to the question of why and how cultural evolution went so much further specifically in our ancestors. How did human culture become open-endedly cumulative?

The hottest lead for what could have set our ancestors apart has for some time been the observation that animal social learning is very poor at preserving the actual know-how behind the functions that traditions serve (e.g. [12,15]). Animal social learning is more about being directed to worthwhile objects of independent learning than it is about copying particular actions being performed (e.g. [12,17]). Clearly, if the underlying behavioural patterns are not even retained, variants of them cannot undergo selection, and cumulative cultural evolution cannot happen. We seem to need a capacity for faithful copying of know-how to get cumulative culture.

But when, how and why did such a capacity arise and prevail in *Homo,* and only in *Homo*? This is not an easy question. Even if cumulative culture is highly adaptive, a closer look at its preconditions reveals that there is a startup problem here. Many pieces must be in place before faithful copying can become even minimally effective as an inheritance system, and it is far from clear why primitive and pre-effective forms of such a capacity would be selected for (see e.g. [15,18]). Basically, if you are not already very good at copying solutions to problems, just coming up with a solution yourself will usually be the better option. And even if you *are* good at copying, re-inventing the solution will seamlessly adapt it to any contingent variations in the setting where it is to be used, which copying will not, so re-invention is also the more robust option.

Placing a definite date on the appearance of this capacity is also hard. Many would place it somewhere around 1.8–2.6 Ma, at the roots of tool-aided cooperative big game carnivory, and of a thenceforth contiguous archaeological record. Things clearly begin to change around this time, and by the end of this timespan we see a dramatic range expansion, evidencing an improved capacity to thrive in many types of habitats, plausibly associated with the advent of culturally enabled active hunting (e.g. [19,20]). It would seem that this ‘Oldowan context’ was where our ancestors decisively began to diverge from a behavioural range we would normally expect from great apes, and it seems fair to guess that culture had something to do with this.

However, attributing faithful copying to early *Homo* may be harder than widely assumed. To the extent that brain organization and relative size had changed at all at this time (e.g. [21,22]), it was in any case not by much, and the actual behaviour evidenced in Oldowan tool technology has been argued to provide weaker evidence for know-how copying and cumulative evolution than has commonly been believed (e.g. [16,23,24]). While the jury is still out on these questions (see e.g. [25,26]), it seems prudent to be conservative when assuming the presence of sophisticated cognitive features in the Oldowan toolmakers.

Unambiguous evidence of faithful copying of know-how in social learning materializes only as late as around half a million years ago (e.g. [16,24,27–30]). However, concluding that this was when *Homo* moved from being just another great ape is less than satisfying: first, since it seems to rob cumulative culture of most of its explanatory power with respect to human evolution up to that point; second, because *something* related to the transition from animal to human culture did seem to happen in this Oldowan context. But if this ‘something’ was not faithful copying via social learning, what else could it possibly have been?

The Social Protocell Hypothesis (SPH; [31–33]) ventures the proposition that cumulative cultural evolution may indeed have arisen in this early *Homo* context, or even in association with considerably earlier hominin carnivory (see e.g. [34,35]), but that it would have happened in a radically different way from what is usually believed. The proposition is that it all started via an overlooked type of *group-level* cultural inheritance that emerges as a fortuitous side-effect of animal culture combined with a certain type of social group behaviour (i.e. the social protocell).

Cultural evolution on this level would have led to cumulative integrated *systems* of animal traditions,^2^ functionally linked into what we may call primitive *institutions* and *cultural lifestyles*.^3^ Their adaptive affordances would be much broader than for stand-alone traditions (see also [36,38,39]). While not permitting sophisticated tools and techniques, institutions would allow complex spatially, temporally and socially distributed systems of simpler practices to emerge around important resources, such as big game carnivory.

On this level, the know-how that must be faithfully copied resides not in the traditions themselves, but in their *structure* as institutional systems. The copying process is based on the splitting of social communities, and is, thereby, independent from sophisticated forms of social learning. The social protocell thereby solves the startup problem without the need to assume the presence of sophisticated cognitive and psychological adaptations in ape-like ancestors. Indeed, the social protocell is argued to be ancient, and active also in present-day *Pan*. The reason why it gave rise to ‘open-ended’ cumulative culture [40] only in *Homo* is argued to be differences in ecological settings, more specifically the presence of large vertebrate carcasses.

Solving this group-level version of the startup problem moreover paves the way for a *later* solution of the original startup problem, in an institutionally structured cultural environment, by large-brained forms of *Homo*, where pre-adaptations for the modern human suite of cognitive and meta-cognitive capabilities for copying and processing culture (see e.g. [41–43]) are considerably easier to imagine (see also [33]).

The SPH follows a familiar evolutionary pathway that is widely considered to have been responsible for most or even all other unequivocal gains in adaptive complexity in natural history (e.g. [44–46]), namely an evolutionary transition in individuality (ETI; see [44,47–53]), where a new group-level unit of selection (or ‘evolutionary individual’) arises. In this case, the new unit of selection (termed a ‘sociont’) would consist of integrated and adapted cultural lifestyles, coextensive with (but *not* identical to) the social limits of underpinning hominin communities (based on face-to-face contacts, and congruous with *Pan* communities today; e.g. [54–56]). While not the first proposition that an ETI (in some shape or form) is responsible for the evolution of our unusual species (see e.g. [57] for a review, as well as other contributions to this issue, e.g. [11,58–60]), the SPH applies the concepts and theory of ETI in some detail, enabling thereby a sustained inquiry, guided by specific interpretations of which entities, levels, relations and processes are proposed to be at work.

We here implement a model that simulates the ETI proposed by the SPH. The question that we pose is, first, whether cumulative cultural evolution on the level of institutions can be demonstrated to robustly appear in a model built to represent the basic entities and mechanisms of the social protocell. Second, we ask whether we can demonstrate some aspect of ‘evolution of evolutionary individuality’ [32], as a proof-of-principle that a cultural ETI is plausible. Third, we ask whether we can demonstrate coevolution between agent and sociont, as the latter emerges as a cultural unit of selection. We will first introduce the SPH and move then to describe the simulation model before we present the results and discuss their implications. The model is described in more detail in supplementary material, appendix SA, and a range of additional runs to test the robustness of the model and chart out further possibilities are presented in supplementary material, appendix SB. See also movies depicting the simulated dynamics at https://youtu.be/WLVa2Ae_vQM and https://youtu.be/GQu9ORywL7s. We begin with a brief outline of the SPH, referring readers to Andersson & Törnberg [31], Davison *et al.* [32] and Andersson & Tennie [33] for more in-depth accounts.

## 2. The social protocell hypothesis

**Figure 1.**
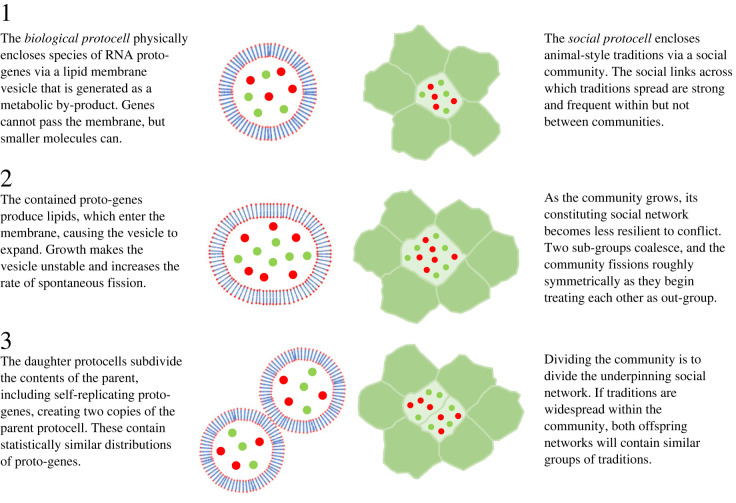
Following Andersson & Törnberg [31] the SPH proposes that social communities impose a group-level lifecycle on collections of traditions and serve the role that lipid membranes do for the biotic protocell. We compare idealized renditions of biotic protocells with their proposed social counterparts.

The term ‘social protocell’ draws a direct parallel with the specific ‘egalitarian’ ETI [61] believed to be responsible for the emergence of cellular life [62–68]. The biotic protocell describes the settings believed to have produced an ETI from proto-genes to simple cells, while the SPH proposes that a ‘social protocell’ became the origin of human culture via the re-appearance of the same abstract entities and relations in a substrate that hardly could have been more different. In both cases, the idea is that a combination of independently explainable adaptations and circumstances coincided to impart a group-level lifecycle via compartmentalization, reproduction, and relatively faithful inheritance of adapted systems of lower-level units (figure 1).

The protocell could be described in general as a coincidental (and thereby suitable for explaining origins, e.g. [51]) ‘pump’ for group selection, providing mechanisms for *alignment of fitness* between the contained lower-level units, and for group-level unit reproduction with *inheritance* of co-adapted combinations between such units.

Alignment of fitness interests (see e.g. [69–71]) results as lower-level units are maintained in proximity, linking thereby their longer-term evolutionary fates (figure 1). ‘Cheating’ lower-level units will be then be more likely to suffer longer-term negative consequences of undermining group-level traits, and to receive benefits from sticking to the cooperative arrangement (‘boomerang factors’, see e.g. [72]). This ‘slows down’ selection on the lower level, leaving space for group-level traits to evolve. Traditions also seem unlikely to be efficient targets of lower-level selection to begin with. For example, while it is easy to see how hominins may readily cheat in a cooperative setting, there would seem to be fewer ways (albeit not impossible)^4^ for traditions to benefit from cheating on institutions. We will not focus on this effect here but assume (at the risk of being wrong,) that the wiggle-room for cheating traditions is sufficiently constrained for adaptive group-level institutions to be possible.

Protocell inheritance is very simple in principle. It follows directly from the expected outcome of splitting a ‘parent’ mix of entities in half being that the two ‘daughter’ mixes will have the same composition in a statistical sense (figure 1). If the traditions maintained in a fissioning community are mastered by a sizeable proportion of its members, and the split is reasonably close to symmetric, we may quite robustly expect that cultural life in the daughter communities will be similar to cultural life in the parent community. So, if some cultural lifestyles cause an elevated rate of growth and splitting, we may expect to see natural selection of more successful variants, at the expense of less successful variant lifestyles.

Simply by the law of large numbers, this inheritance channel may have a reasonably high fidelity, also in the absence of dedicated mechanisms adapted for improving fidelity, and so, at least some degree of cumulative evolution may result on this level. Notably, this does not require that cultural know-how gets copied (let alone with high fidelity) in social learning. As long as *the functions* of the traditions remain stable (which does not appear to be a very strong assumption; see [74,75]), *systems* of such functions may be seen as an emergent type of *institutional* know-how that can be inherited (see also [33]).

The social protocell goes beyond and complements ‘standard’ cultural group selection (e.g. [36,37,76,77]) by accounting for a possible origin of cultural group selection, by being explicit about processes, units, levels and interactions (see e.g. [78]), and, not least, by accounting for how cultural group selection could also have been a bootstrapping process. The prediction that the social protocell may have led to an ETI is based on the potential that group-level selection may act on features that enable *further* group selection (i.e. ‘evolution of evolutionary individuality’, see e.g. [32,48,79,80]^5^, such as *via* the fidelity of inheritance, mechanisms against cheating, and so on).

In other words, while the group-selection-inducing functions of the social protocell *start out* as non-cultural and coincidental, they may (like those of the initially by-product cell membrane in the biotic case) later come under the group selection that they themselves cause, and they may be expanded with additional such functions. The outcome is proposed to be the sociont, as a new cultural unit of selection whose formerly independent components (traditions) become integrated as mere components of an adaptive cultural whole. Notably, the SPH is *not* about group selection and integration of hominins^6^ but about group selection and integration of traditions. The envisioned evolutionary role of *Homo* is in an obligate mutualistic partnership, with the sociont as an emerging cultural unit of selection.

Finally, a note on the origin of the combined traditional activities that we propose could be inherited and selected via the social protocell. Thompson *et al*. [35] proposed that the roots of human carnivory are not necessarily an activity that resembles the pursuit of small vertebrates seen in great apes today (in particular in chimpanzees). On the basis of several lines of evidence, they argue that a pursuit of inside-bone resources, in large bones left behind at predator kill sites, using percussion with locally available rocks, is a more likely starting point. Once established we can imagine that an incipient practice of this sort could be expanded with mutually supporting sub-practices, having to do, for example, with processing, finding and securing access to carcasses, the production and use of tools, and so on.

The state of such a proto-institution could undergo historically path-dependent change if the young tended to follow the older individuals, encountering established sequences of problems and opportunities, to reproduce variations appearing in the combined practice. Even if *some* practice with this function would be likely to appear spontaneously given the presence of the resource (not a culture-dependent trait; see [17]), *particular* variants of such a practice, maintained in particular lineages of communities, may still be highly unlikely to do so. Thereby, if some such persistent variant provided higher, or more secure, returns, the benefit provided to the hominin community could cause it to spread if this caused the community to grow and divide move frequently.

Initially, such a practice may have been similar in style to cultural chimpanzee practices today, for example, nut cracking. The difference would be that the utilization of large animal carcasses can be taken much further with positive returns to investment in sophisticated behaviour. Large animals are also more effective than small animals in this role. Since a large vertebrate carcass is not monopolizable, its returns will be provided to a wide circle of individuals within the social group, without the need to invoke active sharing (e.g. via ‘tolerated theft’, see [81,82]).

## 3. The model

**Figure 2.**
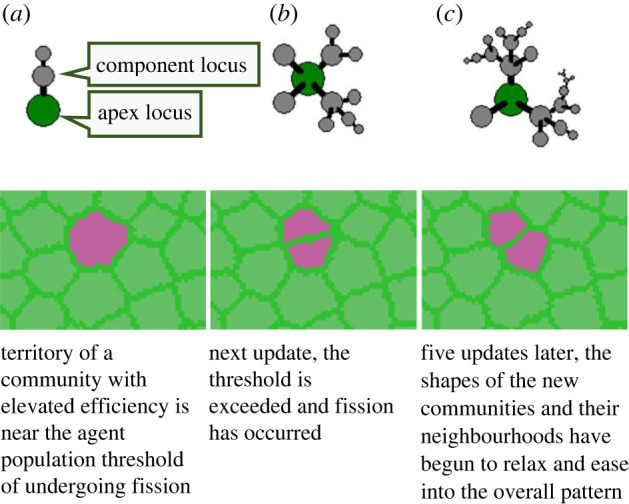
Top row: visualized simulated institutions of increasing complexity. The green node is an apex locus with an external function, and the grey nodes are component loci with internal function subordinated to the function of the apex locus. The complexity classes (mapped to fitness via a fitness function specific to types of apex loci) are, from left to right: 3, 11 and 24. The topology of the network of component loci does not affect fitness in the model as used here. Bottom row: community undergoing fission in a field of communities during a simulation.

The model is an agent-based implementation of the SPH in terms of its components of hominins, group behaviour, social learning and ecological competition for resources and space. See supplementary material, appendix SA for a more in-depth specification of the model. The model has the following entities:

— *Agents.* Agents are born into the social communities of their parents. They go through a lifecycle during which they learn, apply their knowledge, serve as role models for other agents' learning, harvest resources, defend their territory, reproduce, and, finally, die. They have a rank that is determined by lifetime success in obtaining resources.
— *Traditions.* Types of traditions (for specific functions) are referred to as *loci*, while instances of loci (possessed by agents) are referred to as *alleles*.^7^ New loci are invented stochastically. Loci are either directed at a specific external function (such as accessing a resource) or at improving the function of another tradition. We refer to these as *apex* and *component* loci, respectively. Loci, thereby, may be dependent on other loci, representing activities that demand effects of other activities (figure 2, top row). Possessing tradition alleles is costly, and they are socially learned within the community.
— *Institutions.* Dependencies between loci produce hierarchical networks of functions and sub-functions, and an apex locus may have under itself several hierarchical levels of subordinated component loci. For example, if the apex locus represents carnivory, then its tree of dependent component loci represents the distinct sub-activities that constitute that specific cultural strategy for carnivory. Such a tree represents an institution (figure 2, top row), and its efficiency in performing its apex tradition is a function of the complexity of the tree (i.e. the number of tradition loci in the hierarchy). The topology of the tree is fitness-neutral in this setup. The special case of an apex locus without components corresponds to an animal-style tradition and is the evolutionary starting point of institutions.
— *Communities and territories.* A community is a social network of amicable links between agents that collectively defends a territory, and which may split roughly in half when internal conflicts spiral out of control (see [56,83–85]). This is the only agency attributed directly to the community, which otherwise serves as an arena within which agents can be assumed to be in social and physical proximity. For example, the effects and learning opportunities of traditions expressed by agents are available to other agents and traditions within the community, but not outside it. While this strict isolation between communities is an idealization whose effects should be relaxed and investigated, there are reasons to believe that while cultural ideas surely did cross community boundaries, the spread was probably limited without strong and persistent social links between communities (see [31] and references therein). Moreover, the biotic protocell has been found to be robust to, and even benefit from, moderate mixing of heritable material (via fusion) [86].
— *Land*. Land is modelled as a regular grid of geographical cells. Each community controls a contiguous territory consisting of such cells, with collective monopoly on all resources present in it (figure 2, bottom row).
— *Resources and customized effects of culture*. Resources are homogeneous across land and are characterized first by their size and degree of monopolizability, and second by an ‘access function’ that maps the complexity of the access strategy (apex locus of an institution) to a degree of access (efficiency). Generally, the more complex the strategy, the more of the resource can be accessed. However, in most cases, agents face decreasing returns to complexity.^8^ Customized functions with specific effects may also be targeted by apex loci and thereby by institutions, for example, in this study, affecting the rate at which agents learn from other agents.

The main update process updates the model state in synchronous timesteps by running the following sub-processes. Rates of all processes are scaled to a time resolution parameter that determines how many updates are made per unit time (called a ‘year’):

— *Culture*. All agents express their tradition alleles and pay the associated cost. A counter of *expressions* of the different loci is maintained for each community. Institutions are then resolved by ‘populating’ each expressed apex allele with expressions of conditioning alleles in their trees of component loci (figure 2, top row), subtracting from the expression counter for each tradition expression used. Apex alleles for which expressions of all loci in their tree were secured count as a successful performance of the institutional structure and invokes its function.^9^ The effect of a successful institutional performance (e.g. harvested resources, or modified agent features) applies to the agent that performed its apex allele, but see also *Harvesting* below.
— *Harvesting*. Each agent goes about accessing resources armed with the efficiency gained by expressing apex alleles (see *Culture* above). The efficiency without cultural help need not be zero since the access function may return an access larger than zero for a complexity of zero (opportunistic resources, independent learning, etc.). The gain in energy accrues first to the agent expressing the apex allele, but if the resource is not fully monopolizable, all other agents in the community will also obtain a share (e.g. as ‘tolerated theft’ [81,82,87]).
— *Social learning*. Each expression of an allele is also an opportunity for social learning. Agents lacking an allele for a locus that is expressed in the community have a likelihood of learning (gaining an allele) that applies once each time an allele of that locus is expressed by another agent. The learning likelihood associated with each expression may also be biased by the rank of the agent expressing it, and by the relative density of its expression in the community.
— *Territoriality*. Territorial defence is collective and modelled as an ongoing conflict in all perimeter cells. Community strength is simply the population count (over a threshold age). Perimeter cell strength is obtained by abating the community strength from the geometric centre of the community radially outward. Each update, it is determined stochastically whether perimeter cells change owners depending on the local balance of power. Unoccupied cells have a low basic power, representing non-hominin-related risks.
— *Community lifecycle*. Communities exceeding a threshold size split into two daughter communities, with half the territory and half the population going to each. Agents bring along their cultural knowledge. If a community dips below threshold territory or population size, it disperses. Its territory then turns into unoccupied cells, destroying all its remaining agents.^10^
— *Agent dynamics*. In each agent, energy gained is used toward a fixed cost of living, costs for expressing tradition alleles in its possession, and, if activated, cost for increased innate cognitive capacity. The surplus goes to an individual reproduction buffer. Agents reproduce when this buffer exceeds a threshold level. When an agent reproduces, a mate is picked at random in the population for sexual genetic crossover. Genetic information in agents is, however, used only in one of the setups (see below). Otherwise agents are identical. Agents die at a rate that is a function of age and energy level.

The model is seeded with a single centrally placed community of agents, equipped with no cultural knowledge. Without competition the community grows and splits cyclically until the configuration is filled with territories near a competitive equilibrium.^11^ If traditions appear that increase the efficiency of agents in some territory, its equilibrium population density increases, causing it to grow at the expense of neighbouring communities (figure 2, bottom row; see also movies at https://youtu.be/WLVa2Ae_vQM and https://youtu.be/GQu9ORywL7s).

Two kinds of resources are used in our setup. One is a ‘basic resource’ which integrates a variety of opportunistic and highly monopolizable resources that can be efficiently foraged without socially learned strategies. There is a slight improvement gained by targeting it with cultural strategies, but it quickly levels off. The other resource is a model of large animal carcasses. These may not be effectively monopolized, and they may be accessed only with the aid of socially learned strategies. Moreover, the ceiling of how much there is to gain by using more complex cultural strategies is very high. Implicit functions of component loci in an institution targeting this resource should be thought of as representing hierarchies of sub-functions. For example, finding, transporting and processing tool raw materials, or obtaining, protecting, transporting, processing, distributing and storing the resource.

This latter resource is the centre of our focus. Andersson & Törnberg [31] argue that large game carnivory, which began even before the production of sharp stone flakes (the Oldowan industry, beginning *ca* 2.6 Ma [88]), and went on to become the centerpiece of the lifestyle of *Homo*, completes the social protocell by providing an open-ended ‘project’ for cultural specialization that can drive the evolution of more and more complex cultural systems (an IGUT: important, generative and universal tradition; see [31]).^12^

We refer to this resource as *IGUT/carnivory*. Entry-level access to this resource (lone apex locus without dependencies) may be interpreted as a pre-Oldowan exploitation using very simple technology, achievable by a generalized great ape, such as unmodified rocks or bones used for breaking large limb bones (that other animals cannot break open) to access marrow [34]. Once focused on exploiting uncontested remains at predator kill sites, there is a natural path toward obtaining earlier and earlier access to the carcass, with more and more soft tissues left, carrying over to active hunting. Doing so would present the hominins with a ladder of more and more complex situations, with rewards but also new challenges at each step. The Oldowan could then signify the step on this ladder where access to soft outside-bone tissues (requiring cutting implements) was gained. We model this by letting extraction efficiency increase steadily as a function of the complexity of the institution.

The *fidelity* of the heredity process (see *Social protocell* and figure 1) is conceptualized as the likelihood that alleles of all used cultural loci in a parent community survive the formation of a daughter community following a split. It is measured as the frequency with which daughter communities in a split do not experience a decrease in *complexity class*. When we say a community is in a certain complexity class, we refer to the maximum complexity (number of loci in the tree; figure 2, top row) at which a high and stable proportion of its inhabitants successfully perform institutions.

## 4. Results

The exploration begins in a base configuration (BASE) from the standpoint of which we take further exploratory steps where parameters are varied, and features are added. In electronic supplementary material, appendix SB we perform a stability analysis, where the full set of parameters are varied and discussed to verify that the BASE case is representative for the behaviour of the model.

**Figure 3.**
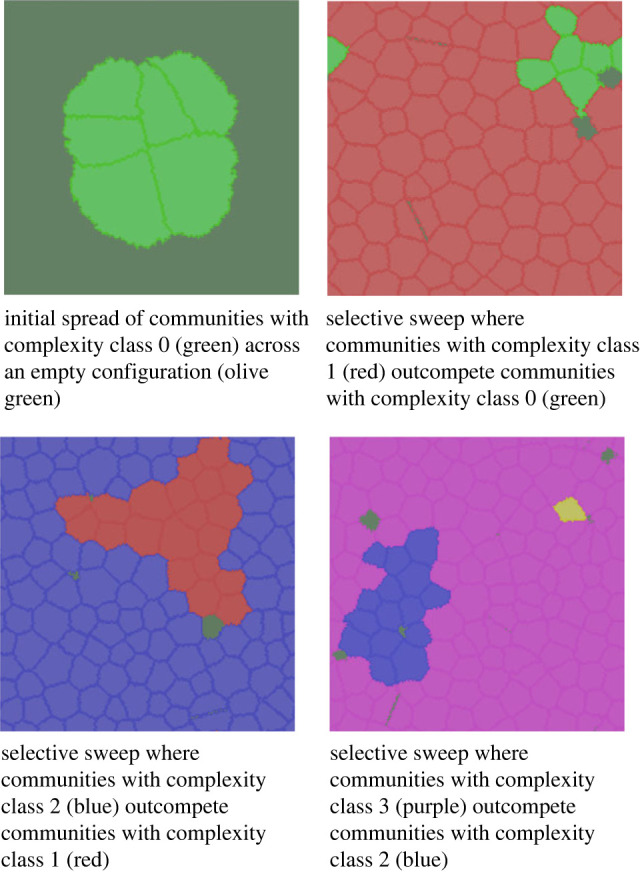
Examples of spatial configurations of community territories during a run using the BASE scenario. Olive green lacunas between territories are territories of communities that have dispersed under pressure. Note how the equilibrium size of communities decreases over time as efficiency of land use increases in higher complexity classes.

Figure 3 illustrates the visual appearance of the simulated dynamics; see also movies at https://youtu.be/WLVa2Ae_vQM and https://youtu.be/GQu9ORywL7s. The population of a community whose agents are better at extracting resources than neighbours will grow. This increases the community's perimeter cells’ strength, also causing its territory to grow. More resources are thereby provided, which stimulates further growth, while the area of weaker neighbours shrinks, placing them under pressure as their resource base dwindles. If they are unable to sustain their population, they are eventually pushed below the population threshold and disperse. The evolutionary dynamic is driven by the increases in efficiency, conveyed by more complex variants of IGUT/carnivory institutions. As a community's population grows it will eventually split, and the institutions that caused the growth will tend to survive these splits, causing successful variants to spread via ‘demic diffusion’ (see e.g. [89–91]).

**Figure 4.**
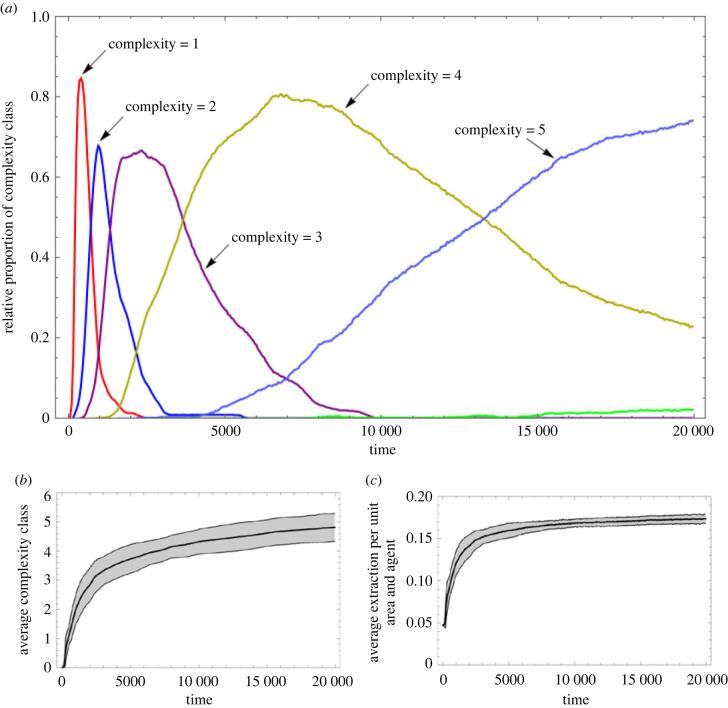
(*a*) The relative proportion of communities in different complexity classes as a function of time. (*b*) Average complexity of IGUT as a function of time. (*c*) Average extraction of resources per unit area and agent as a function of time. We see how the slowdown of the rate of selection sweeps by higher complexity classes (*a*) is matched by markedly decreasing returns to increasing complexity (*b,c*). The BASE case is used, and data are averaged over 100 runs, running across 200 000 updates each (20 000 ‘years ’).

In figure 4*a* we observe averages formed over many histories produced by the model. We see how more and more complex institutions targeting the IGUT resource arise and out-compete incumbent populations of lower complexity classes (since the topology of the institution does not affect fitness, it is sufficient to refer to complexity classes; see §3 *The model* and figure 2, top row.) The replacement is rapid initially but gradually slows down as higher and higher complexity classes are reached. In figure 4*b,c* we observe the evolution of, respectively, average complexity class and harvesting efficiency over time, which indicates that sociont complexity here evolves toward an asymptote.

Complexity, as we see, does not go on increasing indefinitely. Something punishes the higher complexity classes and that ‘something’ is cultural losses in social protocell inheritance. The more complex the transmitted institution gets, the more cultural loci must be continually learned by new generations, which increases the likelihood that some locus will become rare and go missing in a reproduction event (figure 1). This is maladaptive since losing the prevailing carnivory institution infallibly leads to the demise of the community and its supported sociont.^13^

**Figure 5.**
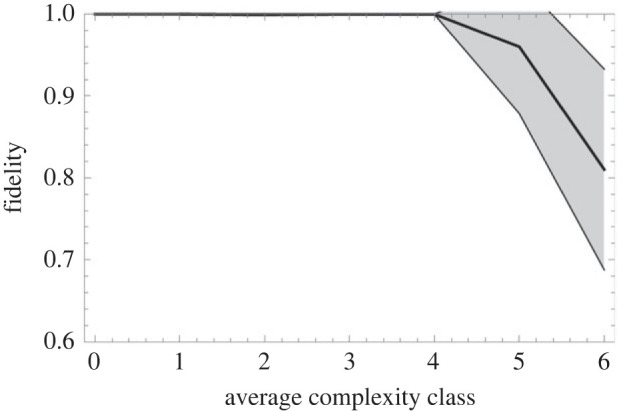
Fidelity of community splitting as an emergent hereditary mechanism for institutions as a function of complexity class. The BASE scenario is used, averaging over 100 runs, and running across 200 000 updates. Shaded interval denotes 1 standard deviation.

Figure 5 shows how the measured fidelity of sociont inheritance decreases dramatically at a point that corresponds well with the value around complexity class 5, where the average complexity asymptote seems to be in the BASE scenario (figure 4). Communities in complexity class 4 reproduce at virtually no rate of loss while communities of complexity class 5 will suffer a reduction in fecundity of *ca* 5%. As we see in figure 4, this does not keep them from slowly taking over the configuration but, at a penalty of *ca* 20%, complexity class 6 remains marginal as its superior efficiency is thereby nullified. This imposes an equilibrium that Andersson *et al.* [92,93,98] refer to as a ‘glass ceiling’, above which increased losses in transmission are not outweighed by gains in efficiency permitted by more complex adaptations (see also [100,101]).

**Figure 6.**
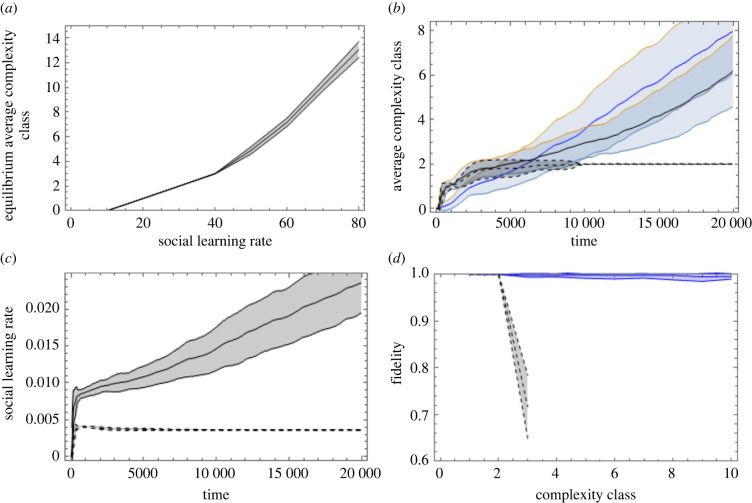
(*a*) Parameter sweep varying the social learning rate parameter (SLR), or otherwise using the BASE scenario (which has SLR = 50.) Achievable sociont-level complexity increases as losses to errors decrease. (*b*) Blue graph shows complexity evolution in the SLR-boosting institution. Black graph shows the carnivory institution. Dashed black graph shows, as reference, the evolution of the carnivory institution, using BASE case without SLR-boosting institution, with SLR = 30 (the value at which SLR begins in the other runs). (*c*) Comparison between the rates of social learning (successful learning events per 1000 opportunities) in the coevolutionary (solid) and reference (dashed) scenarios. (*d*) Comparison between the fidelity of sociont-level inheritance in the coevolutionary (solid) and reference (dashed) cases. Cumulative evolution of the SLR institution stably keeps fidelity close to unity, enabling complexity to keep increasing under selection. Runs average 100 simulations across 200 000 updates (20 000 ‘years’). Shaded interval denotes 1 standard deviation of sampled data points.

The social learning rate (SLR) of agents is controlled by a parameter in the model. Increasing this rate should increase the number of loci learned by each agent, which should increase the fidelity of social protocell inheritance. This, in turn, should increase the evolutionary equilibrium level of sociont complexity. In figure 6*a* we observe the equilibrium complexity class achieved as SLR is varied, confirming that higher values of SLR correspond to higher equilibrium cultural complexity. This indicates that features improving the capacity to engage in social learning (e.g. features pertaining to social tolerance, communication, pedagogy, etc.) could be adaptive by enabling more complex cultural systems that, in turn, are directly adaptive.

To test whether the social protocell is capable in principle of kickstarting a positive cultural evolutionary feedback process, we now add an ‘SLR-boosting’ institution to the mix (an apex locus with that function.) The more complex this institution becomes, the higher the SLR *for those observing alleles expressed by somebody expressing the apex locus of the SLR booster*.^14^ Like for the carnivory institution, component traditions have a cost (the same cost), but in this case no resources are produced—only the effect on rates of social learning.

Figure 6*b* tells us that the simulated social protocell can indeed effect self-reinforcing cumulative coevolution between these two institutional functions. In the BASE case, the carnivory institution rapidly gets stuck at the level of complexity imposed by its rate of social learning (SLR = 30), while the coevolutionary setup keeps evolving more and more complex institutions. Since the IGUT/carnivory institution is adaptive in itself (i.e. it returns resources), its complexity is rapidly driven up by selection from the outset. The SLR-boosting institution has adaptive effects only in the presence of other institutions that it enables, and that yields fitness to the agents, so it begins to increase in complexity somewhat later. As it does, it permits the IGUT/carnivory institution to become more complex, which, in turn, sets off their coevolution.

Increasing the SLR has two effects: (1) it increases the fitness of the carnivory institution by reducing its transmission losses, and, relatedly, (2) it increases the proportion of agents successfully performing the carnivory institution, and thereby the average efficiency. The second effect is likely the most immediate source of fitness for the SLR-boosting institution, and once established on a higher level of complexity, it will also make further complexity increases in the carnivory institution more adaptive.

In figure 6*c*,*d* we see the coevolutionary ETI dynamics from the perspectives of rates of social learning (figure 6*c*) and the fidelity of sociont-level transmission via social community splits (figure 6*d*). The effect of increasing the rate of social learning is that the fidelity of the sociont inheritance mechanism increases. We may speak of it taking shape as an actual adaptation for evolutionary individuality on the sociont level [32]. In other words, this demonstrates that the type of entity that we term a sociont actually does emerge in our simulated system.

Having established that the simulated social protocell can sustain the coevolution of institutions that together boost their own ability to undergo evolution, we have achieved an illustration and proof-of-principle of the potential for an ETI as proposed by the SPH. However, such an ETI would not get very far. The nature of the traditional components of these institutions and their linkages would remain constrained by the innate psychology and cognitive capabilities that were present at the outset, and that thereby would not be specifically adapted for enabling complex institutions.

We will therefore finally ask whether such an innate capacity in the agent can coevolve with the institutions in this simulated setting. For this purpose, we create a three-component ratchet, where the IGUT/carnivory institution ‘pays the bill’, the SLR-boosting institution improves fidelity, and a varying and heritable cognitive feature defines the agents' ability to benefit from complex SLR-boosting institutions.

**Figure 7.**
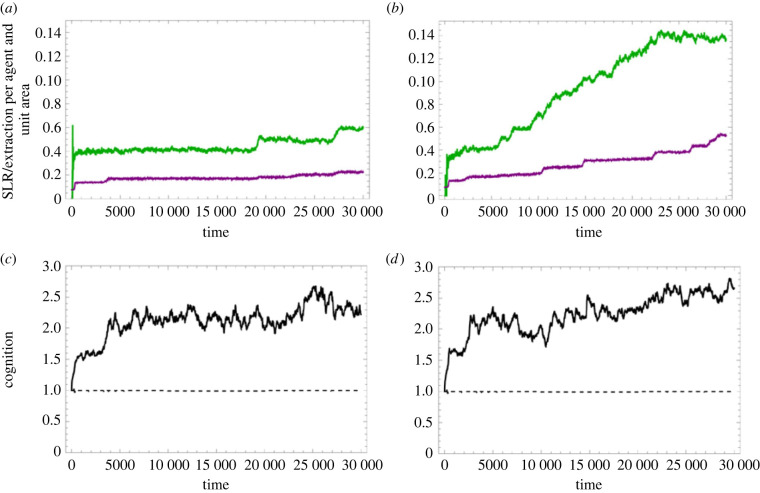
The columns (*a,c*) and (*b,d*) correspond to two runs with different random seed over 300 000 updates (30 000 ‘years’) at a resolution of 500 × 500, with the SLR boost institution and the genetically inherited cognitive capacity active. The BASE case parameters are used, but with SLR = 30. Top row (*a,b*) Evolution of the measured social learning rate (green) and the efficiency of resource utilization (purple). Bottom row (*c,d*) Evolution of the costly cognitive ability to use sophisticated SLR institutions (solid). As a reference, the case where cognition is not coupled with this ability is provided to verify that there is no other source of positive selection for this feature (dashed).

In figure 7 we explore two historical scenarios. Typical simulated histories now become of higher interest than averages over many histories since the runs become highly historically path-dependent.^15^ We may here observe that simulated cognitive capacity (associated with a cost) indeed increases under selection, along with increasing complexity of the SLR-boosting institution, together enabling increasing complexity of the carnivory institution. This demonstrates the potential for coevolution between agents and sociont (third row in figure 7). Please see supplementary material, appendix SC for an in-depth analysis of figure 7.

## 5. Conclusion

We have explored some of the central claims made by the SPH using a simulation model that implements its basic proposed mechanisms. The results indicate that the mechanisms proposed by the SPH do generate the behaviour that they were claimed to generate by Andersson & Törnberg [31] and Davison *et al*. [32]. Our results moreover permit us to specify those claims more precisely and identify ways forward.

The central SPH claims that we have tested may be summarized as follows:

A. Institutions and cultural lifestyles can come to undergo cumulative evolution even if their socially learned components (traditions) do not. The reason is that inheritance of *institutional* know-how, via the social protocell dynamics, can be faithful even if know-how is not inherited in the first place via social learning.
B. Institutions that improve and expand the set of coincidental ‘meta-evolutionary’ functions (such as heredity) that the social protocell provides may also themselves evolve in this way. This paves the way for a cultural ETI where an incipient group-level unit of selection (a sociont) gains more and more evolutionary individuality the more evolutionary individuality it has gained.
C. Genetically inherited features of the agents (here hominins) that maintain the sociont may coevolve with the sociont into an obligate mutualistic partnership.

With regard to claim A, the SPH implies that the cultural channels of the ‘dual-inheritance’ architecture [3,4] are actually *two* hierarchically organized channels: one for ideas, via social learning, and one for systems of such ideas, via the growing-and-splitting dynamics of social networks in which social learning takes place. This is significant since it has turned out to be hard to explain why and how early *Homo* would have shifted from re-invention to faithful imitation of know-how on the level of social learning, which is needed for know-how on that level to undergo cumulative evolution.

The SPH suggests that cumulative evolution of ‘cultural lifestyles’ would have come first, providing a structured and adapted system of ideas, whose component ideas were simple enough to be independently re-invented if motivated and socially cued to do so. From that standpoint, the pressure to assume a capacity for faithful social imitation in early *Homo* is relieved. The alternative SPH story is a much later origin of faithful social imitation, in already culturally structured contexts, maintained by large-brained forms of *Homo*, and at a time when there is actual unequivocal evidence of imitation taking place (approx. 0.5 Ma rather than approx. 2.5 Ma; see [16,24,27–30]). This alternative trajectory is developed as 'trajectory B' by Andersson and Tennie in a forthcoming article [33].

Our results buttress the claim that systems of socially learned ideas can evolve in such a manner. Since know-how is not copied via social learning in the model in the first place, we may be assured that it is not *faithfully* copied either. The traditions are just functions. They do not get more complex, and their performance does not vary. In essence, faithful inheritance remains just as central in our story, but it is first solved on the social protocell level, where weaker assumptions about prior hominin adaptations are sufficient.

Moving to claim B, we have seen that mechanisms that improve and extend the meta-evolutionary functions provided by the social protocell can evolve, even when they are costly and produce only indirect benefits. Similar functions could be aimed for example at detecting and punishing cheating in agents and ideas that undermine the function of institutions, or at the mechanisms and criteria governing community fission. The evolution of such ‘meta-evolutionary’ adaptations of culture (see also e.g. [102–104]) means that the pathway toward an ETI appears to be open in principle.

Considering claim C, we verified that also genetically inherited traits could be added to the system that underwent evolution in our model (figure 7). This feature, moreover, came with a cost but no immediate benefit to the agents. It was adaptive because it was the key to being able to benefit from being born into the cultural context of a sociont that relied on that capability. In principle, this was intended to demonstrate that the pathway toward an obligate mutualistic relation between *Homo* and the sociont as a cultural whole would be open.

The reader is referred to supplementary material, appendix SB for additional provisional findings that may guide the way forward. It may in particular be added that the model was found to be robust to the choice of criterion for community fission. The results thereby do no hinge critically on exactly how this criterion is chosen, which is important since it is not fully understood what, in the end, triggers irreversible fission events on the community level in chimpanzees (which we use as our model species), and, in particular, what the role of the size of the community is in the process [85]. The essential behaviour of the model remained, even if fission just happened randomly at a constant rate regardless of size or any other factor. Supplementary material, appendix SD furthermore contains a discussion of the SPH in the context of some other accounts of a human/cultural ETI represented in this issue.

It may be remarked that a simpler model could have been preferable to a somewhat detailed agent-based model like the one we use. For example, there is an overlap between our model and the highly abstract stochastic corrector model of compartmentalized replicators [65,105]. However, in the cultural case, there is no established theory (corresponding to, for example, biochemistry or genetics) to direct us in abstracting the underpinning dynamics. Such a model would at this point be hard to disentangle in terms of what its features would correspond to empirically in the target system. However, from the standpoint of our agent-based model we are in a better position to make more informed judgements about how to formulate such simpler and more focused models in the future.

Finally, let us stress again that the SPH is purely about a *cultural* ETI. It is not implied, in any way, that *humans* have undergone an ETI. The proposed mutualism between *Homo* and sociont implies none of the signatures that would be expected if *Homo* had been part of an ETI, and the absence of such traits in *Homo*, therefore, does not reflect on the SPH. For example, no particular change in genetic relatedness between humans is predicted, nor any physiological differentiation of humans to specialize in different tasks. On the contrary, as culture became more and more powerful and flexible, physiological specialization to serve narrowly defined tasks (like cell types or social insect castes) could only stand in the way of the more flexible and powerful cultural process of adaptation. The SPH would predict humans to adapt as content-neutral platforms for culture, as flexibly open as possible for adaptive *cultural* differentiation, which is also in accordance with what we see (see e.g. [106,107]). After all, culture makes the difference between *Homo sapiens* as a Middle Palaeolithic hunter and *H.* *sapiens* an astronaut in space.

## Data Availability

Supplementary material is available online [108]. Supplementary movies are available at https://youtu.be/WLVa2Ae_vQM and https://youtu.be/GQu9ORywL7s.

## Endnotes

1 By ‘know-how’ we intend, literally, the knowledge about *how* to go about achieving some desired effect (e.g. cracking nuts or tying one's shoelaces). This is as opposed to what we could call ‘know-what,’ ‘know-when’ or ‘know-why.’ The know-how may be independently re-invented on the basis of knowledge about what to do and motivated by knowledge of why to do it. For example, most can figure out a process for cracking nuts if the need is perceived and then re-apply this know-how when required. One may also study the process implemented by somebody else, in which case the know-how is copied. See e.g. Tennie *et al.* [16].

2 The term *Tradition* is overloaded with several meanings in the literature. We use it to refer to a socially transmitted cognitive and behavioural process that is exercised by a single agent. It is focused on, and motivated by, some main function. ‘Cultural trait’ is another frequently used term. Systems of complementary traditions that collectively serve functions are referred to as *institutions*. Institutions can be distributed socially, temporally and spatially, and gain thereby an emergent and considerably more versatile design space.

3 We use the term ‘institution’ in a sense that is similar to how it has been used by e.g. Smaldino [36] and Richerson *et al*. [37] in the cultural group selection literature.

4 The literature on ‘memes’ contains a wealth of ideas about how ideas may spread in a parasitic fashion (see e.g. [73]). To see how we must keep in mind that traditions cannot *directly* benefit from what they produce (e.g. they don't eat meat) but from somehow enticing agents to learn them. For example, a tradition that produces a more immediate gratification for the learner and practitioner, at the cost of serving less well in the institutional whole, could be an example.

5 If socionts compete based on heritable features, becoming better at supporting and inheriting such features will enable new types of group-level adaptations where the competition cannot follow suit. An evolutionary individual (unit of selection), briefly, is a unit that undergoes evolution by natural selection as a whole.

6 It may or may not entail group selection of hominins.

7 This should not be interpreted as implying a perfect analogy with genetics. We use it because it expediently expresses the difference between functions (loci) and instances of realizations of that function (alleles).

8 Imagine cracking nuts, for example. Once you can reliably crack the nut, the marginal gain from using more sophisticated strategies will be much lower.

9 This step-function loss of fitness may seem dramatic. However, several instances of institutions are typically performed each update by a community, so *fewer* successful executions will result if there is a shortage of agents equipped to perform some needed function. A persistent loss in fitness happens when exactly zero agents possess some needed component locus and the sociont loses it.

10 Only two community splits have been observed in *Pan* in the field [83,85], and both have been roughly symmetric. Observing the process by which splitting unfolds there are also social dynamical reasons for believing that this is generally the case (see e.g. [56]).

11 The equilibrium is never perfect, however, even in the absence of innovation, due to stochasticity in spatial competition and fluctuations in births and deaths in the communities.

12 Important = contributes strongly to fitness; Generative = can be profitably far extended; Universal = available across large contiguous area.

13 Cultural complexity as an equilibrium between selection and rates of learning, error and selection comes as no surprise, and has been widely researched in many forms and contexts, e.g. [15,92–99].

14 So as not to sneak group features in through the back door.

15 This means that individual runs vary substantially and qualitatively so that a very large number of histories are needed to get a smooth average. At the same time, such an average will not resemble any actual time evolution of the system and conceal the details.

## References

[1] Campbell DT. 1960 Blind variation and selective retention in creative thought as in other knowledge processes. Psychol. Rev. **67**, 380-400. (10.1037/h0040373) 13690223

[2] Dawkins R. 1983 Universal Darwinism. In Evolution from molecules to man (ed. DS Bendall), pp. 403-425. Cambridge, UK: Cambridge University Press.

[3] Boyd RT, Richerson PJ. 1985 Culture and the evolutionary process. Chicago, IL: University of Chicago Press.

[4] Cavalli-Sforza LL, Feldman MW. 1981 Cultural transmission and evolution: a quantitative approach. Princeton, NJ: Stanford University Press. 7300842

[5] Whiten A. 2021 The burgeoning reach of social learning and culture in animals' lives. Proc. Annu. Meeting. Cogn. Sci. Soc. **43**, 660-661.

[6] Allen JA. 2019 Community through culture: from insects to whales: how social learning and culture manifest across diverse animal communities. BioEssays **41**, 1900060. (10.1002/bies.201900060) 31631360

[7] Laland KN, Galef BG. 2009 The question of animal culture. Cambridge, MA: Harvard University Press.

[8] Godfrey-Smith P, Calcott B, Sterelny K, Calcott B, Sterelny K. 2011 Darwinian populations and transitions in individuality. In The major transitions in evolution revisited (eds B Calcott, K Sterelny), pp. 65-81. Cambridge, MA: MIT Press.

[9] Dean LG, Vale GL, Laland KN, Flynn E, Kendal RL. 2014 Human cumulative culture: a comparative perspective. Biol. Rev. Camb. Phil. Soc. **89**, 284-301. (10.1111/brv.12053) 24033987

[10] Kempe M, Lycett SJ, Mesoudi A. 2014 From cultural traditions to cumulative culture: parameterizing the differences between human and nonhuman culture. J. Theor. Biol. **359**, 29-36. (10.1016/j.jtbi.2014.05.046) 24928150

[11] Carmel Y. 2023 Human societal development: is it an evolutionary transition in individuality? Phil. Trans. R. Soc. B **378**, 20210409. (10.1098/rstb.2021.0409) 36688399 PMC9869447

[12] Tennie C, Call J, Tomasello M. 2009 Ratcheting up the ratchet: on the evolution of cumulative culture. Phil. Trans. R. Soc. B **364**, 2405-2415. (10.1098/rstb.2009.0052) 19620111 PMC2865079

[13] Tomasello M. 1999 The cultural origins of human cognition. Cambridge, MA: Harvard University Press.

[14] Tomasello M, Kruger A, Ratner H. 1993 Cultural learning. Behav. Brain Sci. **16**, 495-552. (10.1017/S0140525X0003123X)

[15] Boyd RT, Richerson PJ. 1995 Why culture is common, but cultural evolution is rare. Proc. Br. Acad. **88**, 77-93.

[16] Tennie C, Premo LS, Braun DR, McPherron SP. 2017 Early stone tools and cultural transmission: resetting the null hypothesis. Curr. Anthropol. **58**, 652-672. (10.1086/693846)

[17] Tennie C, Hopper LM, van Schaik CP, Hopper LM, Ross SR. 2020 On the origin of cumulative culture: consideration of the role of copying in culture-dependent traits and a reappraisal of the zone of latent solutions hypothesis. In Chimpanzees in context: a comparative perspective on chimpanzee behavior, cognition, conservation, and welfare (eds LM Hopper, SR Ross), Chicago, IL: Chicago University Press.

[18] Shea N. 2009 Imitation as an inheritance system. Phil. Trans. R. Soc. B **364**, 2429-2443. (10.1098/rstb.2009.0061) 19620113 PMC2865078

[19] Fleagle JG, Shea JJ, Grine FE, Baden AL, Leakey RE. 2010 Out of Africa I - the first hominin colonization of Eurasia. Dordrecht, The Netherlands: Springer.

[20] Carotenuto F, Tsikaridze N, Rook L, Lordkipanidze D, Longo L, Condemi S, Raia P. 2016 Venturing out safely: the biogeography of *Homo erectus* dispersal out of Africa. J. Hum. Evol. **95**, 1-12. (10.1016/j.jhevol.2016.02.005) 27260171

[21] Ponce de León MS et al. 2021 The primitive brain of early *Homo*. Science **372**, 165-171. (10.1126/science.aaz0032) 33833119

[22] Du A, Wood B, Cole J, McNabb J, Grove M, Hosfield R. 2020 Brain size evolution in the hominin clade. In Landscapes of human evolution (eds J Cole, J McNabb, M Grove, R Hosfield), pp. 9-17. Oxford, UK: Archaeopress Publishing.

[23] Tennie C, Braun DR, Premo LS, McPherron SP, Haidle MN, Conard NJ, Bolus M, Haidle MN, Conard NJ. 2016 The island test for cumulative culture in the paleolithic. In The nature of culture (eds MN Haidle, NJ Conard, M Bolus), pp. 121-133. Dordrecht, The Netherlands: Springer Science.

[24] Corbey R, Jagich A, Vaesen K, Collard M. 2016 The Acheulean handaxe: more like a bird's song than a Beatles' tune? Evol. Anthropol. **25**, 6-19. (10.1002/evan.21467) 26800014 PMC5066817

[25] Stout D, Rogers MJ, Jaeggi AV, Semaw S. 2019 Archaeology and the origins of human cumulative culture: a case study from the earliest Oldowan at Gona, Ethiopia. Curr. Anthropol. **60**, 309-340. (10.1086/703173)

[26] Toth N, Schick K. 2018 An overview of the cognitive implications of the Oldowan Industrial Complex. Azania **53**, 3-39. (10.1080/0067270X.2018.1439558)

[27] Corbey R. 2020 Baldwin effects in early stone tools. Evol. Anthropol. **29**, 237-244. (10.1002/evan.21864) 32835429 PMC7693078

[28] Vaesen K, Houkes W. 2020 Is human culture cumulative? Curr. Anthropol. **62**, 218-238. (10.1086/714032)

[29] Acerbi A, Tennie C. 2016 The role of redundant information in cultural transmission and cultural stabilization. J. Comp. Psychol. **130**, 62-70. (10.1037/a0040094) 26881945 PMC4763254

[30] Snyder AWD, Reeves JS, Tennie C. 2022 Early knapping techniques do not necessitate cultural transmission. Sci. Adv. **8**, eabo2894. (10.1126/sciadv.abo2894) 35857472 PMC9258951

[31] Andersson C, Törnberg P. 2019 Toward a macroevolutionary theory of human evolution: the social protocell. Biol. Theory **14**, 86-102. (10.1007/s13752-018-0313-y)

[32] Davison DR, Andersson C, Michod RE, Kuhn SL. 2021 Did human culture emerge in a cultural evolutionary transition in individuality? Biol. Theory **16**, 213-236. (10.1007/s13752-021-00382-x)

[33] Andersson C, Tennie C. Submitted. Zooming out the microscope on cumulative cultural evolution – a ‘trajectory B’ from animal to human culture. *Hum. Social Sci. Commun*.

[34] Pobiner BL. 2020 The zooarchaeology and paleoecology of early hominin scavenging. Evol. Anthropol. **29**, 68-82. (10.1002/evan.21824) 32108400

[35] Thompson JC, Carvalho S, Marean CW, Alemseged Z. 2019 Origins of the human predatory pattern: the transition to large-animal exploitation by early hominins. Curr. Anthropol. **60**, 1-23. (10.1086/701477)

[36] Smaldino PE. 2014 The cultural evolution of emergent group-level traits. Behav. Brain Sci. **37**, 243-254. (10.1017/S0140525X13001544) 24970399

[37] Richerson PJ et al. 2016 Cultural group selection plays an essential role in explaining human cooperation: a sketch of the evidence. Behav. Brain Sci. **39**, e30. (10.1017/S0140525X1400106X) 25347943

[38] Sasaki T, Biro D. 2017 Cumulative culture can emerge from collective intelligence in animal groups. Nat. Commun. **8**, 1. (10.1038/s41467-016-0009-6) 28416804 PMC5399285

[39] Buskell A, Enquist M, Jansson F. 2019 A systems approach to cultural evolution. Palgrave Commun. **5**, 131. (10.1057/s41599-019-0343-5)

[40] Borg JM, Buskell A, Kapitany R, Powers ST, Reindl E, Tennie C. 2022 Evolved open-endedness in cultural evolution: a new dimension in open-ended evolution research. *ArXiv*, 2203.13050v2. (10.48550/arXiv.2203.13050) 37253238

[41] Dunstone J, Caldwell CA. 2018 Cumulative culture and explicit metacognition: a review of theories, evidence and key predictions. Palgrave Commun. **4**, 145. (10.1057/s41599-018-0200-y)

[42] Heyes CM. 2016 Who knows? Metacognitive social learning strategies. Trends Cogn. Sci. **20**, 204-213. (10.1016/j.tics.2015.12.007) 26778808

[43] Shea N, Boldt A, Bang D, Yeung N, Heyes CM, Frith CD. 2014 Supra-personal cognitive control and metacognition. Trends Cogn. Sci. **18**, 186-193. (10.1016/j.tics.2014.01.006) 24582436 PMC3989995

[44] Carmel Y, Shavit A. 2020 Operationalizing evolutionary transitions in individuality. Proc. R. Soc. B **287**, 20192805. (10.1098/rspb.2019.2805) PMC7031674 32019441

[45] Marcot JD, McShea DW. 2007 Increasing hierarchical complexity throughout the history of life: phylogenetic tests of trend mechanisms. Paleobiology **33**, 182-200. (10.1666/06028.1)

[46] Stewart JE. 2014 The direction of evolution: the rise of cooperative organization. Biosystems **123**, 27-36. (10.1016/j.biosystems.2014.05.006) 24887200

[47] Maynard-Smith J, Szathmáry E. 1995 Major transitions in evolution. New York, NY: W.H. Freeman Press.

[48] Michod RE. 1999 Darwinian dynamics: evolutionary transitions in fitness and individuality. Princeton, NJ: Princeton University Press.

[49] Michod RE. 2007 Evolution of individuality during the transition from unicellular to multicellular life. Proc. Natl Acad. Sci. USA **104**(Suppl.1), 8613-8618. (10.1073/pnas.0701489104) 17494748 PMC1876437

[50] Leigh EG. 2010 The evolution of mutualism. J. Evol. Biol. **23**, 2507-2528. (10.1111/j.1420-9101.2010.02114.x) 20942825

[51] Clarke E. 2014 Origins of evolutionary transitions. J. Biosci. **39**, 303-317. (10.1007/s12038-013-9375-y) 24736161

[52] Hanschen ER, Shelton DE, Michod RE. 2015 Evolutionary transitions in individuality and recent models of multicellularity. In Evolutionary transitions to multicellular life (eds I Ruiz-Trillo, AM Nedelcu), pp. 165-187. Dordrecht, The Netherlands: Springer Science and Business Media.

[53] Szathmáry E. 2015 Toward major evolutionary transitions theory 2.0. Proc. Natl Acad. Sci. USA **112**, 10 104-10 111. (10.1073/pnas.1421398112) 25838283 PMC4547294

[54] Layton R, O'Hara S, Bilsborough A. 2012 Antiquity and social functions of multilevel social organization among human hunter-gatherers. Int. J. Primatol. **33**, 1215-1245. (10.1007/s10764-012-9634-z)

[55] Dunbar RIM. 1993 Coevolution of neocortical size, group size and language in humans. Behav. Brain Sci. **16**, 681-735. (10.1017/S0140525X00032325)

[56] Moffett MW. 2013 Human identity and the evolution of societies. Hum. Nat. **24**, 219-267. (10.1007/s12110-013-9170-3) 23813244

[57] Waring TM, Wood ZT. 2021 Long-term gene–culture coevolution and the human evolutionary transition. Proc. R. Soc. B **288**, 20210538. (10.1098/rspb.2021.0538) PMC8170228 34074122

[58] Dor D. 2023 Communication for collaborative computation: two major transitions in human evolution. Phil. Trans. R. Soc. B **378**, 20210404. (10.1098/rstb.2021.0404) 36688385 PMC9869436

[59] Kish Bar-On K, Lamm E. 2023 The interplay of social identity and norm psychology in the evolution of human groups. Phill. Trans. R. Soc. B **378**, 20210412. (10.1098/rstb.2021.0412) PMC9869443 36688389

[60] Prentiss AM, Laue C, Gjesfjeld E, Walsh MJ, Denis M, Foor TA. 2023 Evolution of the Okvik/Old Bering Sea culture of the Bering Strait as a major transition. Phil. Trans R. Soc. B **378**, 20210415. (10.1098/rstb.2021.0415) 36688384 PMC9869439

[61] Queller DC. 1997 Cooperators since life began. Q. Rev. Biol. **72**, 184-188. (10.1086/419766)

[62] Gánti T. 1975 Organization of chemical reactions into dividing and metabolizing units: the chemotons. Biosystems **7**, 15-21. (10.1016/0303-2647(75)90038-6) 1156666

[63] Gánti T. 1997 Biogenesis itself. J. Theor. Biol. **187**, 583-593. (10.1006/jtbi.1996.0391) 9299301

[64] Michod RE. 1983 Population biology of the first replicators: on the origin of the genotype, phenotype and organism. Am. Zool. **23**, 5-14. (10.1093/icb/23.1.5)

[65] Szathmáry E. 1986 The eukaryotic cell as an information integrator. Endocytobiosis Cell Res. **3**, 113-132.

[66] Szathmáry E, Demeter L. 1987 Group selection of early replicators and the origin of life. J. Theor. Biol. **128**, 463-486. (10.1016/S0022-5193(87)80191-1) 2451771

[67] Szathmáry E, Maynard-Smith J. 1995 The major evolutionary transitions. Nature **374**, 227-232. (10.1038/374227a0) 7885442

[68] Norris V, Raine DJ. 1998 A fission-fusion origin for life. Orig. Life Evol. Biosph. **28**, 523-537. (10.1023/A:1006568226145) 9742727

[69] Michod RE, Viossat Y, Solari CA, Hurand M, Nedelcu AM. 2006 Life-history evolution and the origin of multicellularity. J. Theor. Biol. **239**, 257-272. (10.1016/j.jtbi.2005.08.043) 16288782

[70] Folse HJ II, Roughgarden J. 2010 What is an individual organism? A multilevel selection perspective. Q. Rev. Biol. **85**, 447-472. (10.1086/656905) 21243964

[71] Niklas KJ, Newman SA. 2013 The origins of multicellular organisms. Evol. Dev. **15**, 41-52. (10.1111/ede.12013) 23331916

[72] Mesterton-Gibbons M, Dugatkin LA. 1992 Cooperation among unrelated individuals: evolutionary factors. Q. Rev. Biol. **67**, 267-281. (10.1086/417658)

[73] Schlaile MP, Veit W, Boudry M, Dopfer K, Nelson RR, Potts J, Pyka A. In press. Memes. In Routledge handbook of evolutionary economics (eds K Dopfer, RR Nelson, J Potts, A Pyka). London, UK: Routledge.

[74] Mercader J, Panger M, Boesch C. 2002 Excavation of a chimpanzee stone tool site in the African rainforest. Science **296**, 1452-1455. (10.1126/science.1070268) 12029130

[75] Mercader J, Barton H, Gillespie J, Harris J, Kuhn S, Tyler R, Boesch C. 2007 4,300-year-old chimpanzee sites and the origins of percussive stone technology. Proc. Natl Acad. Sci. USA **104**, 3043-3048. (10.1073/pnas.0607909104) 17360606 PMC1805589

[76] Boyd RT, Richerson PJ. 1990 Group selection among alternative evolutionarily stable strategies. J. Theor. Biol. **145**, 331-342. (10.1016/S0022-5193(05)80113-4) 2232821

[77] Henrich J. 2004 Cultural group selection, coevolutionary processes and large-scale cooperation. J. Econ. Behav. Organ. **53**, 143-162. (10.1016/S0167-2681(03)00094-5)

[78] Whiten A, Erdal D. 2016 Clarifying the time frame and units of selection in the cultural group selection hypothesis. Behav. Brain Sci. **39**, 45. (10.1017/S0140525X15000291) 27561447

[79] Buss LW. 1987 The evolution of individuality. Princeton, NJ: Princeton University Press.

[80] Radzvilavicius AL, Blackstone NW. 2018 The evolution of individuality revisited. Biol. Rev. **93**, 1620-1633. (10.1111/brv.12412) 29575407

[81] Blurton Jones NG. 1984 A selfish origin for human food sharing: tolerated theft. Ethol. Sociobiol. **5**, 1-3. (10.1016/0162-3095(84)90030-X)

[82] Winterhalder B. 1996 A marginal model of tolerated theft. Ethol. Sociobiol. **17**, 37-53. (10.1016/0162-3095(95)00126-3)

[83] Furuichi T. 1987 Sexual swelling, receptivity, and grouping of wild pygmy chimpanzee females at Wamba, Zaïre. Primates **28**, 309-318. (10.1007/BF02381014)

[84] Goodall J. 1986 The chimpanzees of Gombe: patterns of behavior. Cambridge, MA: Belknap Press.

[85] Feldblum JT, Manfredi S, Gilby IC, Pusey AE. 2018 The timing and causes of a unique chimpanzee community fission preceding Gombe's ‘Four-Year War’. Am. J. Phys. Anthropol. **166**, 730-744. (10.1002/ajpa.23462) 29566432

[86] Vig-Milkovics Z, Zachar I, Kun Á, Szilágyi A, Szathmáry E. 2019 Moderate sex between protocells can balance between a decrease in assortment load and an increase in parasite spread. J. Theor. Biol. **462**, 304-310. (10.1016/j.jtbi.2018.11.020) 30471297

[87] Stevens JR, Gilby IC. 2004 A conceptual framework for nonkin food sharing: timing and currency of benefits. Anim. Behav. **67**, 603-614. (10.1016/j.anbehav.2003.04.012)

[88] Semaw S et al. 2003 2.6-Million-year-old stone tools and associated bones from OGS-6 and OGS-7, Gona, Afar, Ethiopia. J. Hum. Evol. **45**, 169-177. (10.1016/S0047-2484(03)00093-9) 14529651

[89] Mulder MB, Nunn CL, Towner MC. 2006 Cultural macroevolution and the transmission of traits. Evol. Anthropol. **15**, 52-64. (10.1002/evan.20088)

[90] Guglielmino CR, Viganotti C, Hewlett B, Cavalli-Sforza LL. 1995 Cultural variation in Africa: role of mechanisms of transmission and adaptation. Proc. Natl Acad. Sci. USA **92**, 7585-7589. (10.1073/pnas.92.16.7585) 11607569 PMC41384

[91] Lycett SJ, Collard M, McGrew WC. 2009 Cladistic analyses of behavioural variation in wild *Pan troglodytes*: exploring the chimpanzee culture hypothesis. J. Hum. Evol. **57**, 337-349. (10.1016/j.jhevol.2009.05.015) 19762070

[92] Andersson C. 2011 Paleolithic punctuations and equilibria: did retention rather than invention limit technological evolution? PaleoAnthropology **2013**, 243-259. (10.4207/PA.2011.ART55)

[93] Andersson C. 2013 Fidelity and the emergence of stable and cumulative sociotechnical systems. PaleoAnthropology **2013**, 88-103. (10.4207/PA.2013.ART81)

[94] Henrich J. 2004 Demography and cultural evolution: how adaptive cultural processes can produce maladaptive losses: the Tasmanian case. Am. Antiq. **69**, 197-214. (10.2307/4128416)

[95] Rendell L, Boyd RT, Enquist M, Feldman MW, Fogarty L, Laland KN. 2011 How copying affects the amount, evenness and persistence of cultural knowledge: insights from the social learning strategies tournament. Phil. Trans. R. Soc. B **366**, 1118-1128. (10.1098/rstb.2010.0376) 21357234 PMC3049108

[96] Laland KN, Rendell L. 2013 Cultural memory. Curr. Biol. **23**, R736-R740. (10.1016/j.cub.2013.07.071) 24028955

[97] Andersson C, Read DW. 2016 The evolution of cultural complexity: not by the treadmill alone. Curr. Anthropol. **57**, 261-286. (10.1086/686317)

[98] Andersson C, Törnberg P. 2016 Fidelity and the speed of the treadmill: the combined impact of population size, transmission fidelity, and selection on the accumulation of cultural complexity. Am. Antiq. **81**, 576-590. (10.1017/S0002731600004017)

[99] Lewis HM, Laland KN. 2012 Transmission fidelity is the key to the build-up of cumulative culture. Phil. Trans. R. Soc. B **367**, 2171-2180. (10.1098/rstb.2012.0119) 22734060 PMC3385684

[100] Eigen M, Schuster P. 1977 The hypercycle. A principle of natural self-organization. Naturwissenschaften **64**, 541-565. (10.1007/BF00450633) 593400

[101] Szathmáry E, Maynard-Smith J. 1997 From replicators to reproducers: the first major transitions leading to life. J. Theor. Biol. **187**, 555-571. (10.1006/jtbi.1996.0389) 9299299

[102] Heyes CM. 2018 Cognitive gadgets. Cambridge, MA: Belknap Press.

[103] Ardila A. 2018 Historical development of human cognition. Singapore, Republic of Singapore: Springer Nature.

[104] Acerbi A, Ghirlanda S, Enquist M. 2014 Regulatory traits: cultural influences on cultural evolution. In Evolution, complexity and artificial life (eds S Cagnoni, M Mirolli, M Villani), pp. 135-147. Berlin, Germany: Springer.

[105] Grey D, Hutson V, Szathmáry E. 1995 A re-examination of the stochastic corrector model. Proc. R. Soc. B **262**, 29-35. (10.1098/rspb.1995.0172)

[106] Lewontin RC. 1972 The apportionment of human diversity. Evol. Biol. **6**, 381-398.

[107] Foley RA, Lahr MM. 2011 The evolution of the diversity of cultures. Phil. Trans. R. Soc. B **366**, 1080-1089. (10.1098/rstb.2010.0370) 21357230 PMC3049104

[108] Andersson C, Czárán T. 2023 The transition from animal to human culture—simulating the social protocell hypothesis. Figshare. (10.6084/m9.figshare.c.6340224) PMC9869448 36688383

